# Defective Proteasome Delivery of Polyubiquitinated Proteins by Ubiquilin-2 Proteins Containing ALS Mutations

**DOI:** 10.1371/journal.pone.0130162

**Published:** 2015-06-15

**Authors:** Lydia Chang, Mervyn J. Monteiro

**Affiliations:** 1 Center for Biomedical Engineering and Technology, University of Maryland School of Medicine, Baltimore, Maryland, United States of America; 2 Department of Anatomy and Neurobiology, University of Maryland School of Medicine, Baltimore, Maryland, United States of America; Children's Hospital of Pittsburgh, University of Pittsburgh Medical Center, UNITED STATES

## Abstract

Ubiquilin proteins facilitate delivery of ubiquitinated proteins to the proteasome for degradation. Interest in the proteins has been heightened by the discovery that gene mutations in *UBQLN2* cause dominant inheritance of amyotrophic lateral sclerosis (ALS). However, the mechanisms by which the mutations cause ALS are not known. Here we report on the underlying defect of ubiquilin-2 proteins containing ALS-linked mutations in affecting proteasome-mediated degradation. We found that overexpression of ubiquilin-2 proteins containing any one of five different ALS mutations slow degradation of Myc, a prototypic proteasome substrate. Examination of coprecipitating proteins indicated that the mutant proteins are generally capable of binding polyubiquitinated proteins, but defective in binding the proteasome. GST-pulldown studies revealed that many of the mutants bind weaker to the S5a subunit of the proteasome, compared with wild type (WT) ubiquilin-2 protein. The results suggest the mutant proteins are unable to deliver their captured cargo to the proteasome for degradation, which presumably leads to toxicity. Quantification of cell death is consistent with this idea. Measurement of protein turnover further indicated the mutant proteins have longer half-lives than WT ubiquilin-2. Our studies provide novel insight into the mechanism by which ALS-linked mutations in *UBQLN2* interfere with protein degradation.

## Introduction

Amyotrophic lateral sclerosis (ALS) is a rapidly progressive neurodegenerative disorder that is associated with loss of upper and lower motor neurons [1]. The disease typically strikes in the fifth decade of life with classical symptoms being the loss of voluntary movements, including speaking, swallowing, walking and breathing. A devastating feature of the disease is the debilitating and rapid deterioration of symptoms that invariably leads to fatality within two to five years after diagnosis. There is currently no treatment to halt or cure ALS.

Major insight into ALS is emerging from understanding the genetic underpinnings of the disease. Although the vast majority of ALS cases are sporadic, about 10% can be traced to inheritance of mutant genes. Genetic studies have identified mutations in ~20 different genes by their linkage to familial ALS [2–4]. Classification of the genes according to their function indicates two large clusters, those involved in protein homeostasis (proteostasis) and RNA metabolism, suggesting that defects in these two systems are a major trigger for ALS [3, 4]. However, the remaining genes are involved in a multitude of cellular functions suggesting ALS can be triggered by alteration in a broad variety of cellular targets.

One of the genes mutated in ALS that functions in proteostasis is *UBQLN2*, an intronless gene located on the X chromosome. Discovery that mutations in the *UBQLN2* cause ALS was first reported in five families with dominant inheritance of the disease [5]. The five families were each found to carry single point mutations that were predicted to encode nonsynonymous substitutions of different proline residues (P497H, P497S, P506T, P509S and P525S mutations) in an unusual PXX repeat motif of unknown function located in the central domain of the 624 amino acids (aa) long ubiquilin-2 protein. Subsequent to this discovery several additional mutations in *UBQLN2* have been reported [6–8]. However, the mechanisms by which any of the *UBQLN2* mutations cause ALS is not known, although some initial studies of some of them suggest the encoded proteins might interfere with proteasomal degradation [5, 9, 10]. How the mutations interfere with proteasomal degradation is not clear as all of the *UBQLN2* mutations described so far map in the central domain of the encoded ubiquilin-2 protein, but not in its surrounding functional ubiquitin-like (UBL) and ubiquitin-associated (UBA) domains, located at the amino-terminus and carboxyl-terminus, respectively [11–13]. The UBA domain has the capacity to bind polyubiquitinated proteins whereas the UBL domain helps deliver the captured polyubiquitinated proteins to the proteasome by binding the S5a subunit of the proteasome [14–16]. Here we investigated the molecular basis for how mutant ubiquilin-2 proteins carrying different ALS mutations affect proteasomal degradation. We show that the mutant proteins are not compromised in polyubiquitin binding, but instead most defective in binding the proteasome.

## Materials and Methods

### Cell Culture and Cell Death Assays

HeLa cells were grown in DMEM supplemented with 10% FBS (Sigma, St. Louis, MO) at 37°C with 5% CO_2_. For the cell death, turnover, and immunoprecipitation experiments, cells were plated at equal densities and transfected with equal amounts of plasmid cDNAs using Lipofectamine LTX (Life Technologies, Grand Island, NY) for 5 to 6 hours (hrs) using the protocol provided by the manufacturer. For quantification of cell death, cultures, 20 post-transfection, were incubated with Hoechst 33342 at a concentration of 1μg/mL for 15 minutes (min) and then 3 fluorescence images from random areas of the dishes were captured on a Zeiss 100 inverted microscope using a DAPI filter and Achromat 10X objective lens. The images were analyzed using iVision-Mac software (BioVision Technologies, Exton, PA) and the percentage of dying cells was quantified by counting the proportion of cells that had abnormally bright fluorescence and abnormal nuclear morphology relative to the total number of cells on the dish [17]. Cells in mitosis were counted as normal. At least 300 cells were counted per plate, per experiment, repeated at least three times. We verified the constructs induce cell death using the Trypan Blue exclusion assay [18]. The transfection efficiency was determined by staining a coverslip that was added to the dishes for expression of the HA-tagged ubiquilin-2 constructs. We routinely obtained >95% transfection efficiency in the experiments.

### Immunoprecipitations

HeLa cell cultures plated at similar densities were either mock transfected or transfected with WT or mutant ubiquilin-2 cDNAs. 20 hrs after transfection the cultures were washed in ice-cold 1x PBS and the cells were scraped from the dishes in RIPA buffer (0.2% NP40, 50mM Tris pH 7.5, 150mM NaCl, 2mM EDTA, 1mM Pefabloc, 5uM leupeptin). They were then sheared by repeated passage through a 25-gauge needle and clarified by centrifugation at 5000 rpm for 5 min. The supernatants were removed and added to 20 μl of RIPA buffer equilibrated Protein A/G Dynabeads (Life Technologies, Grand Island, NY) and rotated for 30 min to remove non-specific binding. The supernatants were recovered following separation of the beads with a magnet and transferred to a fresh tube containing 40 μl of RIPA buffer equilibrated Protein A/G Dynabeads that were previously preincubated with 5μL of rabbit anti-HA antibody for 1.5 hr. The mixture was incubated at 4°C with gentle rotation. The beads were then recovered with a magnet and washed five times with RIPA buffer. SDS-sample buffer was then added to the beads and the mixture was then heated at 100°C for 5 min. Equal portions of the supernatants were then separated by SDS-PAGE and immunoblotted for the proteins mentioned.

### Cycloheximide-chase experiments

The turnover of HA-tagged ubiquilin-2 and endogenous Myc protein was measured by immunoblotting equal amounts of protein lysate that were collected at various time intervals, as indicated in the figures, from HeLa cultures to which cycloheximide was added to a final concentration 100 μM to block new protein synthesis [19].

### Proteasome activity assays

Proteasome activity (chymotrypsin-like activity) was measured using the 20S proteasome activity assay kit (Millipore), which uses *N*-succinyl-Leu-Leu-Val-Try-AMC (7-amino-4-methycoumarin) as a fluorogenic substrate. In these assays, equal amounts of protein contained in microsomes prepared from HEK293-CD3cells that were either mock transfected or transfected with siRNAs against erasin or ubiquilin-1/2 were incubated with the fluorogenic substrate with and without the proteasome inhibitor lactacystin [20]. After 1 hr incubation of the reactions at 37°C, the fluorescence (380 nm excitation/460 nm emission) was measured using a fluorescent plate reader (SpectraMax Gemini; MDS Analytical Technologies), and the proteasome activity reported was calculated by subtracting the fluorescence reading of the reaction incubated with lactacystin from that lacking the inhibitor. The linearity of the fluorescence detection of the plate reader was established using increasing amounts of 7-amino-4-methycoumarin (Enzo Life Sciences, Inc., Farmingdale, NY).

### Immunoblotting

Protein lysates were prepared by washing cell cultures 3 times with ice-cold PBS followed by addition of a solution composed of 0.5% SDS, 0.5% NP40, 0.5% sodium N-lauroylsarcosine, 50 mM Tris pH 6.8, 150 mM NaCl, 20 mM EDTA, 1 mM EGTA, 25 mM sodium fluoride, 1 mM sodium orthovanadate, 1 mM Pefabloc (AEBSF, Roche, Indianapolis, IN), 1 mM leupeptin, and 1 mM aprotonin) [21]. The lysates were then sheared by repeated passage through a 25-gauge needle at least 15 times. The protein concentration of the lysates was determined by the bicinchonic acid assay (BCA) (Thermo Fisher Scientific, Waltham, MA) and mixtures prepared containing equal amount of protein in standard SDS/gel loading buffer. The mixtures were heated for 5 min at 100°C and equal amount of the samples were separated by SDS-PAGE. The separated proteins in the gels were then transferred onto a 0.45 μm PVDF membrane (Millipore, Billerica, MA) for 3hr at 200mA using the Mini-Trans Blot cell system (BioRad, Berkeley, CA) and the membranes probed by immunoblotting with appropriate primary and secondary antibodies. The following primary antibodies were used: mouse monoclonal antibodies to HA (Sigma-Aldrich, St Louis, MO), Myc (9E10 prepared in-house), ubiquitin (#sc8017, Santa Cruz Biotechnology, Inc., Dallas, TX) and rabbit antibodies to HA, GST (all generated in-house), and goat antibodies to actin (Santa Cruz Biotechnology). Binding of primary antibodies was detected by incubation with appropriate secondary horseradish peroxidase-conjugated antibodies using the SuperSignal West Pico system (Thermo Fisher Scientific, Rockford IL) and the chemiluminescence signal was captured using the Fluoro-Chem M imager (Protein Simple, Santa Clara, CA). The following secondary antibodies were used: goat anti-mouse, goat anti-rabbit and rabbit anti-goat (Pierce Biotechnology, Rockford, IL). The intensity of different bands was quantified using AlphaView software (Protein Simple).

### Cloning and expression of recombinant proteins

Ubiquilin-2 proteins encoding either WT or each of the five different ALS mutations mentioned were expressed in eukaryotic or prokaryotic cells as either HA- or GST-tagged proteins, respectively. The five ALS mutations described by [5] were introduced into the cDNA encoding full-length human ubiquilin-2 by site-directed mutagenesis using the procedure as described previously [22]. The resulting cDNAs were fully sequenced and verified to encode only the desired mutations. For eukaryotic expression, cDNAs encoding the complete open reading frame (ORF) of ubiquilin-2 was cloned in-frame downstream of the HA tag using the CMV-HA expression vector (Clontech Laboratories, Inc., Mountain View, CA). For prokaryotic expression the cDNAs were cloned in-frame downstream of GST using the pGST/His T1 vector (GE Healthcare Bio-Sciences Corp, Piscataway, NJ). All the expression constructs were found to encode the correct size polypeptides by immunoblotting.

GST-fusion proteins were purified from *Escherichia coli* BL21(DE3) that had been transformed with the expression plasmids and induced with 0.4 mM IPTG for 4 hrs at 37°C using a standard protocol. The proteins were dialyzed overnight in 20mM Tris pH 8.0, 150mM NaCl, 10% glycerol buffer and adjusted for equal protein content of the full-length polypeptide by Coomassie blue staining. The HIS-tagged human S5a protein was expressed in bacteria following cloning of the entire open reading frame (377 aa) in the pET-21a (Novagen, Billerica, MA) vector. The plasmids were transformed into *E*. *coli* BL21(DE3) and protein expression was induced with 1.0 mM IPTG for 4 hrs at 37°C. Bacteria were lysed by sonication and frozen at -20°C until needed.

### HIS-tag protein purification

Bacterial pellets containing induced HIS-S5a protein was thawed and resuspended in a solution containing 50mM Tris pH 8.0 and 10% sucrose. The suspension was incubated with fresh lysozyme to 0.2 mg/l and left on ice for 30 min. The bacteria were lysed by addition of NP40 to a final concentration of 0.1%. Following 10 min of incubation on ice the lysates was sonicated at 30% output two times for 2 min using a Branson Sonifier 450 (Branson Ultrasonics, Danbury, CT), and then centrifuged at 20,000 rpm for 30 min at 4°C in the 70Ti rotor. The supernatant was collected and incubated with Ni-NTA agarose beads (Qiagen Inc., Valencia, CA), for 4 hrs at 4°C with gentle rotation. The mixture was then loaded onto a 10mL column and washed with 3 column volumes of wash buffer (50mM Tris pH 8.0, 350mM NaCl, 10% glycerol, 30mM imidiazole) and 2 column volumes of wash buffer lacking NaCl and then the bound HIS-tagged proteins were eluted with 50mM Tris pH 8.0,10% glycerol, 250mM imidazole. The eluted proteins were dialyzed against 20mM Tris pH 8.0, 150mM NaCl, 10% glycerol buffer and frozen in small aliquots at -80°C.

### GST protein purifications and GST-pull-down assays

Bacteria pellets from the GST inductions were lysed and clarified as described for the His-purification procedure. The resulting supernatants were incubated with glutathione-agarose beads (Sigma-Aldrich) for 2 hrs at 4°C with gentle rotation. The mixture was poured onto a column and after extensive washing the GST proteins were eluted from the column with 20mM Tris 8.0, 10% glycerol, 1mM EDTA, and 10mM glutathione. The purified GST-fusion proteins were dialyzed in buffer composed of 20mM Tris pH 8.0, 150mM NaCl and 10% glycerol for 3 hrs at 4°C, then aliquoted in small amounts and stored until needed at -80°C. Protein concentration was determined using the BCA reagent for each purified GST-tagged ubiquilin-2 fusion protein. Equal amount of each protein was then separated by SDS-PAGE and the gel stained with Coomassie Blue to ensure the protein used in the assays were of similar purity and for estimation of protein loading. The intensity of full-length GST-ubiquilin-2 band at ~100 kDa was used to gage protein loading. Equal amounts of each GST purified protein (4 μg) were incubated with the same amount of HIS-S5a protein (2 μg) in 800μL 2% Chaps buffer (20mM Tris pH 8.0, 150mM NaCl, 2mM MgCl_2_, 10% glycerol, 2.0% Chaps). 70μL of equilibrated glutathione agarose beads were added and the proteins were incubated for 2 hrs rotating at room temperature. The mixture was centrifuged again at 5000 rpm for 5 min and the supernatant was discarded. The beads were washed four times with 1 x PBS with 2% Tween-20 and 200 mM KCL and one time with 1 X PBS, re-suspended in the SDS sample buffer and heated for 5 min at 100°C. Equal volumes of the pulldown assays were separated by SDS-PAGE and proteins were detected for S5a and GST protein pulldown. The ratio of S5a pulldown relative to the GST-fusion protein was calculated for each sample.

### Statistical Analysis

Experiments were performed a minimum of three times. Student’s t-test and ANOVA tests were performed using GraphPad Prism software (GraphPad Software Inc, San Diego, CA). Data where a significant difference (p<0.05%) was found is shown by an *.

## Results

### Ubiquilin-2 proteins containing ALS mutations have longer half-lives

**Fig 1:**
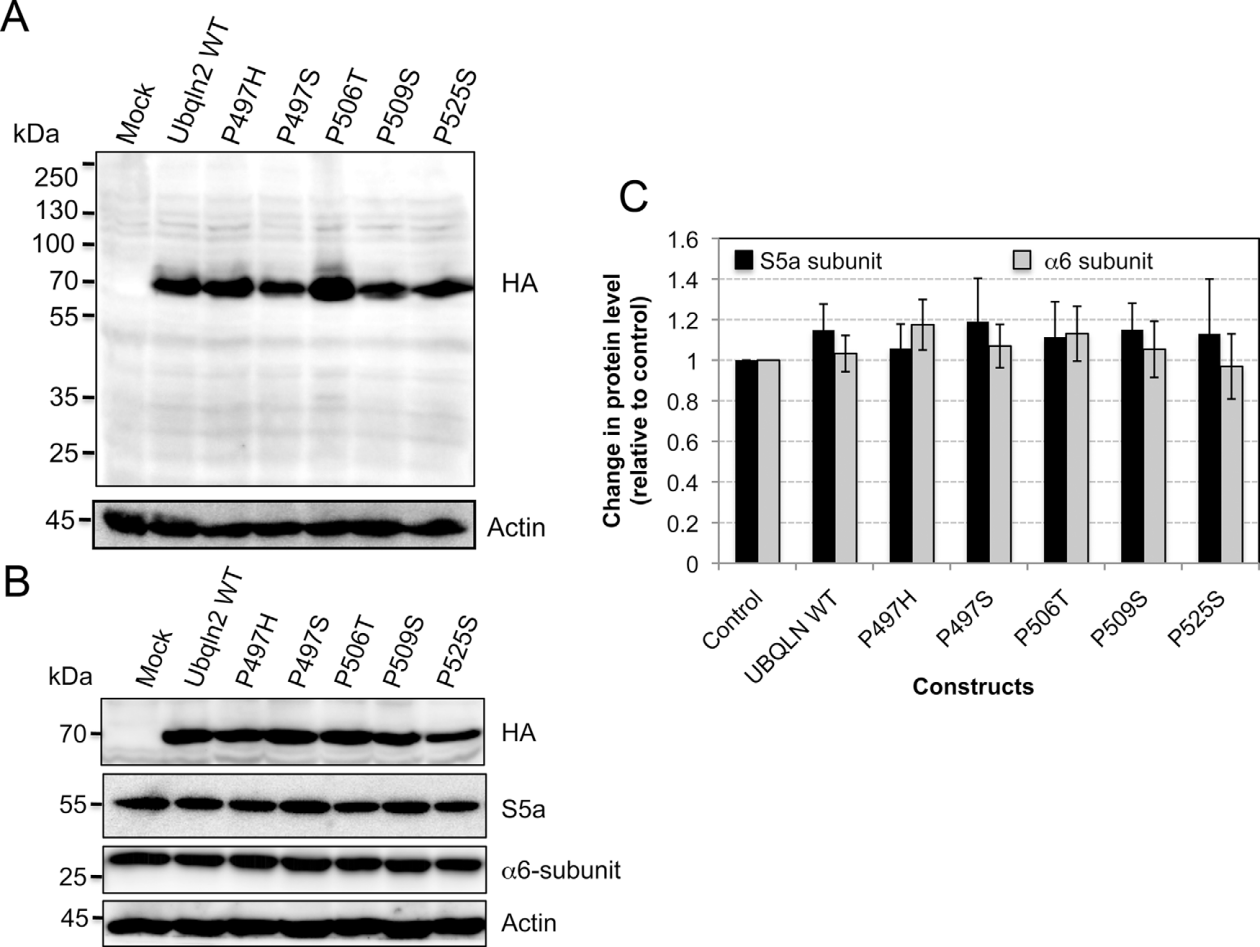
Immunoblot showing similar expression of HA-tagged ubiquilin-2 proteins encoding WT and the five different ALS mutations in HeLa cells. (A) HeLa cell cultures were either mock transfected or transfected with identical amounts of cDNAs encoding WT ubiquilin-2 or each of the five different ALS mutations shown. Equal amounts of protein lysate from the transfections, were resolved by SDS-PAGE, transferred to PVDF membrane, and immunoblotted with HA or actin antibodies. (B) Similar to A, but this time the lysates were also probed for S5a and the alpha 6-subunits of the proteasome. (C) One-way ANOVA analysis revealed no statistical change in the levels of the S5a and the alpha 6-subunit in cells transfected with the ubiquilin-2 constructs compared to that in control mock transfected cells. Errors bars show standard error of the mean.

To determine if ubiquilin-2 proteins carrying ALS-linked mutations are inherently different from the wild type (WT) protein, and/or from one another, we compared the half-lives of the proteins following expression in HeLa cells, suspecting they might be different. Accordingly, we expressed cDNAs encoding full-length WT human ubiquilin-2 and each of the five mutations described by Deng et al. [5] as N-terminal HA-tagged proteins in HeLa cells and measured the turnover of the proteins by cycloheximide-chase experiments. Immunoblots of the protein lysates from the transfected cells using an anti-HA antibody revealed only one reactive band of the predicted size for full-length ubiquilin-2 polypeptide. We did not detect any additional fragment(s) that might have been the counterpart of the C-terminal ubiquilin-2 fragment reported previously [23]. The proteins were expressed at similar levels, although slight differences were noted (Fig 1A). The expressed proteins were found by biochemical fractionation experiments to have similar distributions in the cell [18]. We also analyzed whether overexpression of the ubiquilin-2 proteins affects accumulation of proteasome components, but found no statistical alteration in the levels of either the S5a or the alpha 6-subunit, two random proteasome subunits we selected for examination (Fig 1B and 1C).

**Fig 2:**
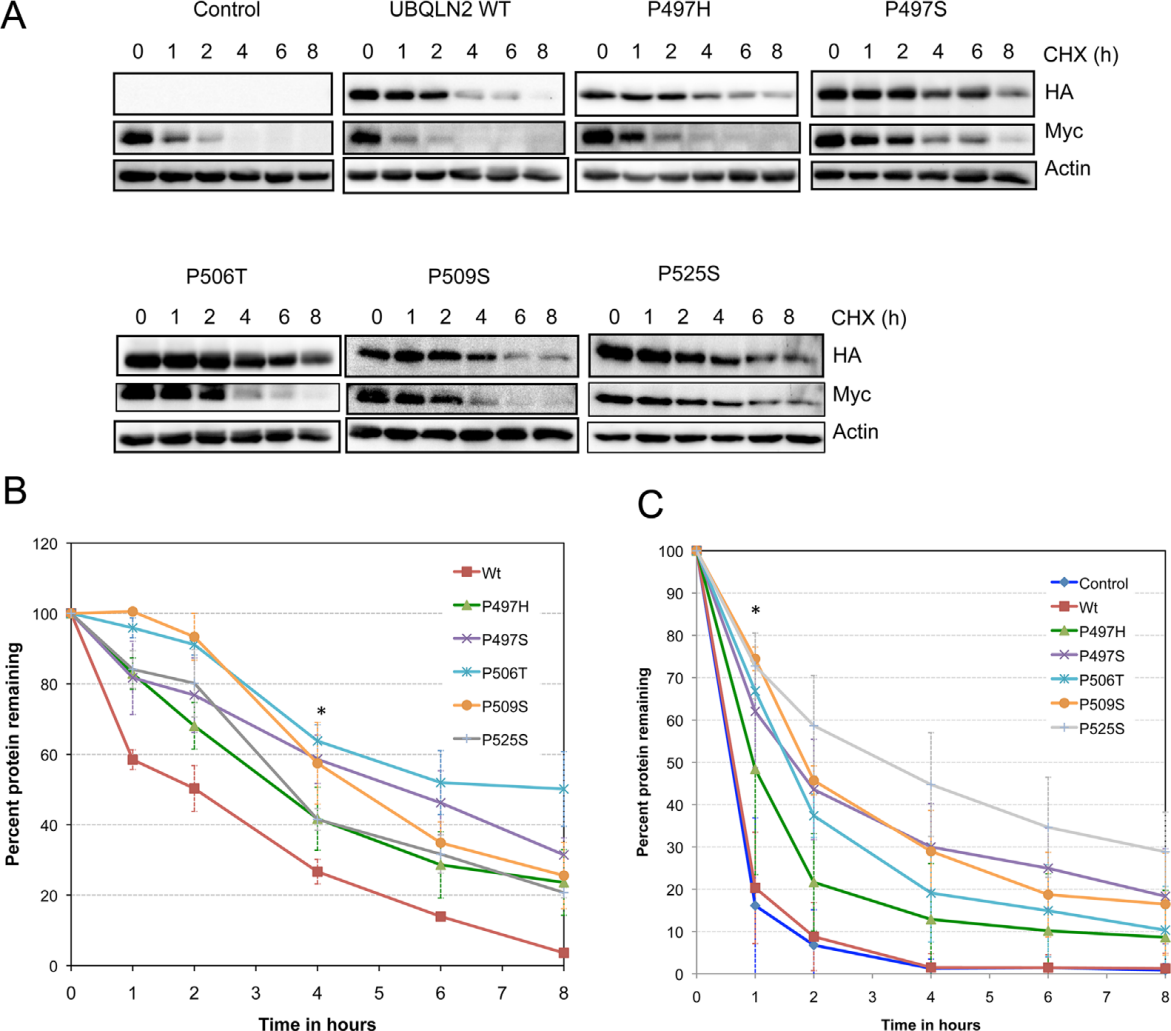
Ubiquilin-2 proteins containing ALS mutation have increased stability and they slow degradation of Myc protein. (A) Similar to Fig 1, except that 1 hrs following transfection the cells were trypsinized from the dishes and replated equally into six plates. 20 hrs later, cycloheximide (CHX) was added for the periods indicated and equal amounts of protein lysates from the cultures were immunoblotted for HA, Myc, and actin. (B) Graph illustrating differences in turnover of the different HA-tagged ubquilin-2 proteins. The turnover of all of the mutants, with the exception of P497H, was slower than WT ubiquilin-2 at the 4 hr time point (* p = <0.05). (C) Similar to B, but showing quantification of Myc protein turnover. Myc protein turnover was significantly slower at the 1 hr time point for cells transfected with all of the mutants, but not WT ubiquilin-2 cDNA (p = 0.05).

We next measured the turnover of the expressed ubiquilin-2 proteins using cycloheximide-chase experiments. Analysis of their turnover indicated all of the ubiquilin-2 mutants had longer half-lives than the WT protein (Fig 2A and 2B). Two-way ANOVA analysis of the data indicated that all of the mutant proteins, except for the P497H mutant, had statistically longer half-lives compared with WT ubiquilin-2 protein (evident at the 4 hr time point. p<0.05). Further analysis indicated there was no statistical difference between the stability of the mutants with each other.

### Overexpression of all five ubiquilin-2 mutations slow Myc protein degradation

Because ubiquilin protein function in protein degradation [24, 25] we next examined whether overexpression of wild type ubiquilin-2 and each of the five ubiquilin-2 mutants affects Myc protein degradation. Myc protein was examined because it is subject to rapid ubiquitin-dependent proteasomal degradation [26]. Also, we wanted to monitor turnover of an endogenous protein rather than an artificial reporter. 2-way ANOVA quantification of the data revealed that overexpression of all five ubiquilin-2 mutants significantly slowed Myc protein turnover at the 1 hr time point compared with mock transfected cells (p< 0.05) (Fig 2A and 2C). Importantly, Myc protein stability was not altered by overexpression of WT ubiquilin-2, indicating the change brought about by the mutants is not from simple overexpression of ubiquilin-2 (Fig 2A and 2C).

### ALS ubiquilin-2 mutants can capture polyubiquitinated proteins efficiently

**Fig 3:**
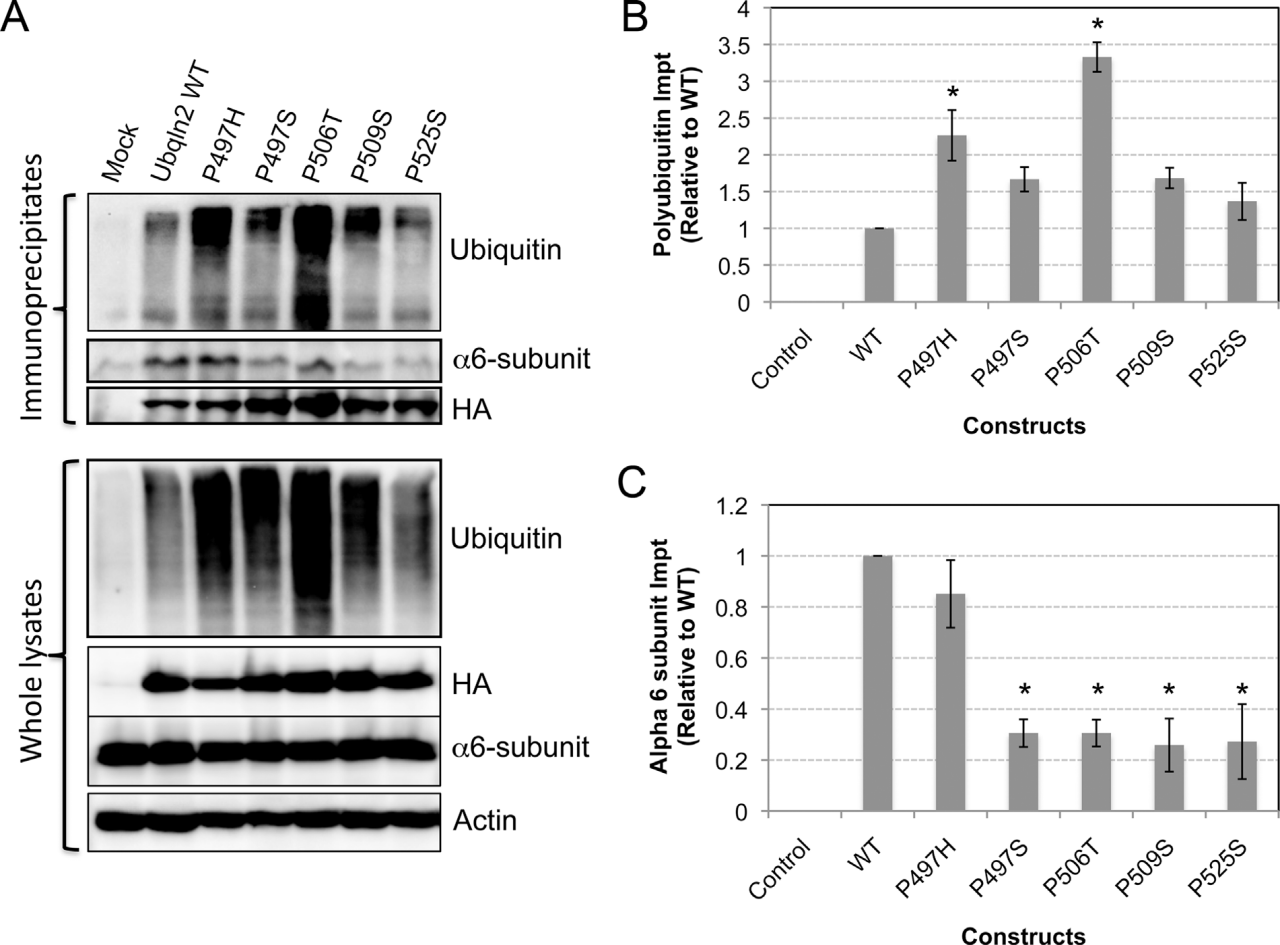
Ubiquilin-2 proteins containing ALS mutations bind polyubiquitinated proteins, but are defective in binding the proteasome. (A) HeLa cells were transfected with the HA-tagged ubiquilin constructs similar to Fig 1. 24 hrs after the transfection, lysates were made from each culture and divided into two equal portions. One set was used to immunoprecipitate the expressed HA-tagged ubiquilin-2 proteins using a polyclonal anti-HA antibody. Equal amounts of the precipitates were then examined for immunoprecipitation of the HA-tagged ubquilin-2 proteins and for co-immunoprecipitation of ubiquitinated proteins (anti-ubiquitin) and the alpha 6-subunit of the proteasome. Equal amounts of protein from the remaining half of the lysates were immunoblotted for ubiquitin, HA, alpha 6-subunit, and actin. (B) Graph showing quantification of the polyubiquitin signal that was co-immunoprecipitated relative to the amount of HA-tagged ubiquilin-2 immunoprecipitated in each lane. This ratio was then normalized to that of WT ubiquilin-2. Errors bars show standard error of the mean. The * indicated the two mutants that bound significantly more polyubiquitin proteins (p<0.05). (C) Similar to B, but showing analysis for the alpha 6-subunit. The * indicates the four mutants that bound significantly less alpha 6-subunit compared with WT ubiquilin-2 (p<0.05).

Because ubiquilin proteins shuttle polyubiquitinated proteins to the proteasome for degradation we next examined whether the reduction in Myc protein turnover induced by the U*BQLN2* mutants stems from a general failure of the mutants to bind polyubiquitinated proteins. To examine this possibility we immunoprecipitated the ubiquilin-2 proteins with an antibody against HA, which we used to tag each of the expressed proteins, and examined the precipitates for the presence of ubiquitinated proteins by immunoblotting (Fig 3). The specificity of the immunoprecipitation was assessed by conducting a control immunoprecipitation of cells not transfected with any HA expression construct. The immunoblots revealed a higher abundance of ubiquitinated proteins that coimmunoprecipitated with all of the HA-tagged WT and ubiquilin-2 mutants, compared with the mock control (Fig 3A). Quantification of the ubiquitin immunoreactivity relative to HA-tagged protein brought down in each lane revealed more polyubiquitinated proteins coimmunoprecipitated with two of the mutants, P497H and P506T, than with WT ubiquilin-2 (Fig 3B). The other mutants (P497S, P509S and P525S) also all brought down more polyubiquitinated proteins than the WT protein, but the difference was not statistically significant (Fig 3B). We considered several possibilities that might account for the general increase in association of polyubiquitinated proteins with the mutants. Either the mutants bind the polyubiquitinated proteins with higher affinity or the mutants are defective in delivery of the polyubiquitinated proteins to the proteasome for degradation. Another unlikely, but remote possibility is that the mutants increase polyubiquitination of proteins, which then associate with the mutants. Based on the known function of ubiquilin proteins we considered the former two possibilities to be the more likely reason.

### The ubiquilin-2 mutants are defective in proteasome binding

To test the idea that the ubiquilin-2 mutants are defective in binding the proteasome, we repeated the immunoprecipitations but this time probed the precipitates for the alpha 6-subunit of the proteasome, which is a component of the 20S core particle of the proteasome [27, 28]. The amount of alpha 6-subunit that was coimmunoprecipitated was normalized to the amount of HA-tagged protein that was immunoprecipitated for each construct (Fig 3A). The quantification revealed that all of the mutants, except the P497H mutant, bound significantly less alpha 6-subunit than WT ubiquilin-2 protein (Fig 3C). The results indicate a general trend whereby most of the *UBQLN2* mutants bind less efficiently to the proteasome.

### In vitro binding experiments confirm the ubiquilin-2 mutants are defective in proteasome binding

**Fig 4:**
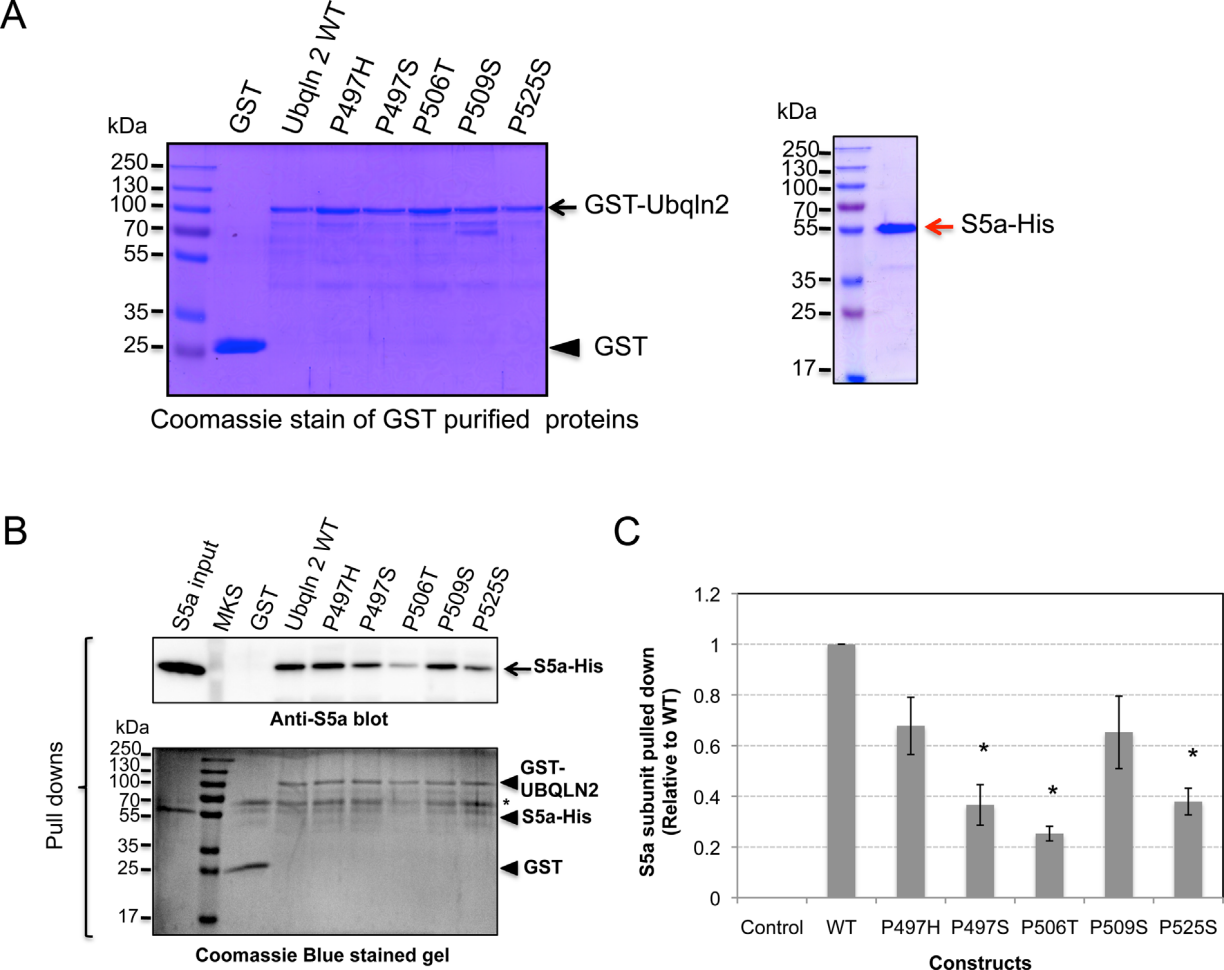
In vitro binding studies showing ubiquilin-2 proteins carrying ALS mutations have reduced binding with the S5a subunit of the proteasome compared with WT ubiquilin-2 protein. (A) Left. SDS-PAGE gel stained with Coomassie Blue stained gels showing the different GST purified proteins (right) and the His-tagged S5a subunit of the proteasome that were used in the binding experiments. (B) Equal amounts of GST or GST-ubiquilin-2 proteins were incubated with a fixed amount of His-S5a for 2 hours. After incubation the beads were washed and the amount of His-S5a that was bound to the beads was analyzed by immunoblotting. Equal amounts of the eluate from the binding experiments were separated by SDS-PAGE together with 1/10 of the input S5a-His protein (Lane 1) and either immunoblotted for S5a, or a parallel gel stained with Coomassie Blue. The positions of the different proteins are marked with arrows. The band marked with an * corresponds to BSA that was added as a blocking agent. (C) Quantification of the GST-pulldown results normalized to WT ubiquilin-2 protein. Errors bars show standard error of the mean. The * signifies a statistical reduction in the binding of S5a by several of the GST-fusion ubiquilin-2 mutants compared to ubiquilin-2 WT (p = <0.05).

Previous studies have shown the UBL domain of ubiquilin docks with the S5a subunit of the proteasome [14], suggesting the likely mechanism by which ubiquilin proteins facilitate delivery of polyubiquitinated proteins captured by its UBA domain for proteasomal degradation. To determine if the reduction in proteasome binding was from a failure of the ubiquilin-2 mutants to bind the S5a subunit, we conducted GST pulldown assays. Accordingly, GST-fusion proteins encoding either WT ubiquilin-2 or each of the five mutations were purified together with His-tagged human S5a protein (Fig 4A). Coomassie staining revealed that all the GST-ubiquilin-2 proteins were of similar purity (Fig 4A) reducing the likelihood that any difference in S5a-His binding stemmed from differences in their purity. The pulldown assays revealed that the GST-fusion proteins carrying each of the five *UBQLN2* mutations bound significantly less S5a-His compared with the fusion protein encoding WT ubiquilin-2 (Fig 4B). As expected, the control GST alone bound only trace amounts of S5a-His. Quantification of the pulldowns indicated that GST-ubiquilin-2 proteins containing all five ALS mutations have reduced binding to the S5a subunit of the proteasome (Fig 4C). One-way ANOVA analysis of the data indicated that reduction in binding was significant for the P497S, P506T and P525S mutations, but not the P497H and P509S mutations. The data supports the idea that ubiquilin-2 proteins carrying ALS mutations are defective in delivery of ubiquitinated substrates to the proteasome for degradation.

### Overexpression of ubiquilin-2 proteins carrying ALS mutations does not affect proteasome activity

**Fig 5:**
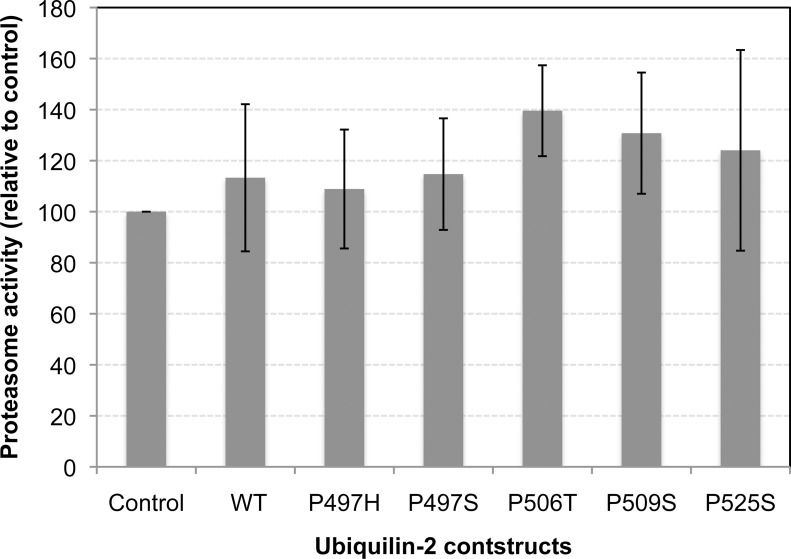
Overexpression of the ubiquilin-2 mutants does not alter proteasome activity. HeLa cell cultures were transfected similar to Fig 1. 24 hrs after transfection lysates were made from the cultures and equal amounts of proteins from each were analyzed for proteasome activity. The transfection efficiency in the cultures was measured by staining a coverslip introduced in the dishes and found to be >95%. Graph showing there was negligible difference in proteasome activity in cell cultures that were mock transfected or transfected with WT or ALS mutant ubiquilin-2 cDNAs.

Because the main defect of the ubiquilin-2 mutants is in delivery of substrates to the proteasome for degradation we predicted that overexpression of the ubiquilin-2 mutants in cells would not affect proteasome activity. Accordingly we measured proteasome activity (chymotrypsin activity) of equal amounts of protein in lysates prepared from HeLa cells that were either mock transfected or transfected with WT or the ubiquilin-2 mutant cDNAs (Fig 5). Consistent with our prediction, we found proteasome activity was not significantly changed by overexpression of either the WT or mutant ubiquilin-2 proteins.

### Overexpression of ubiquilin-2 proteins carrying ALS mutations increase cell death

**Fig 6:**
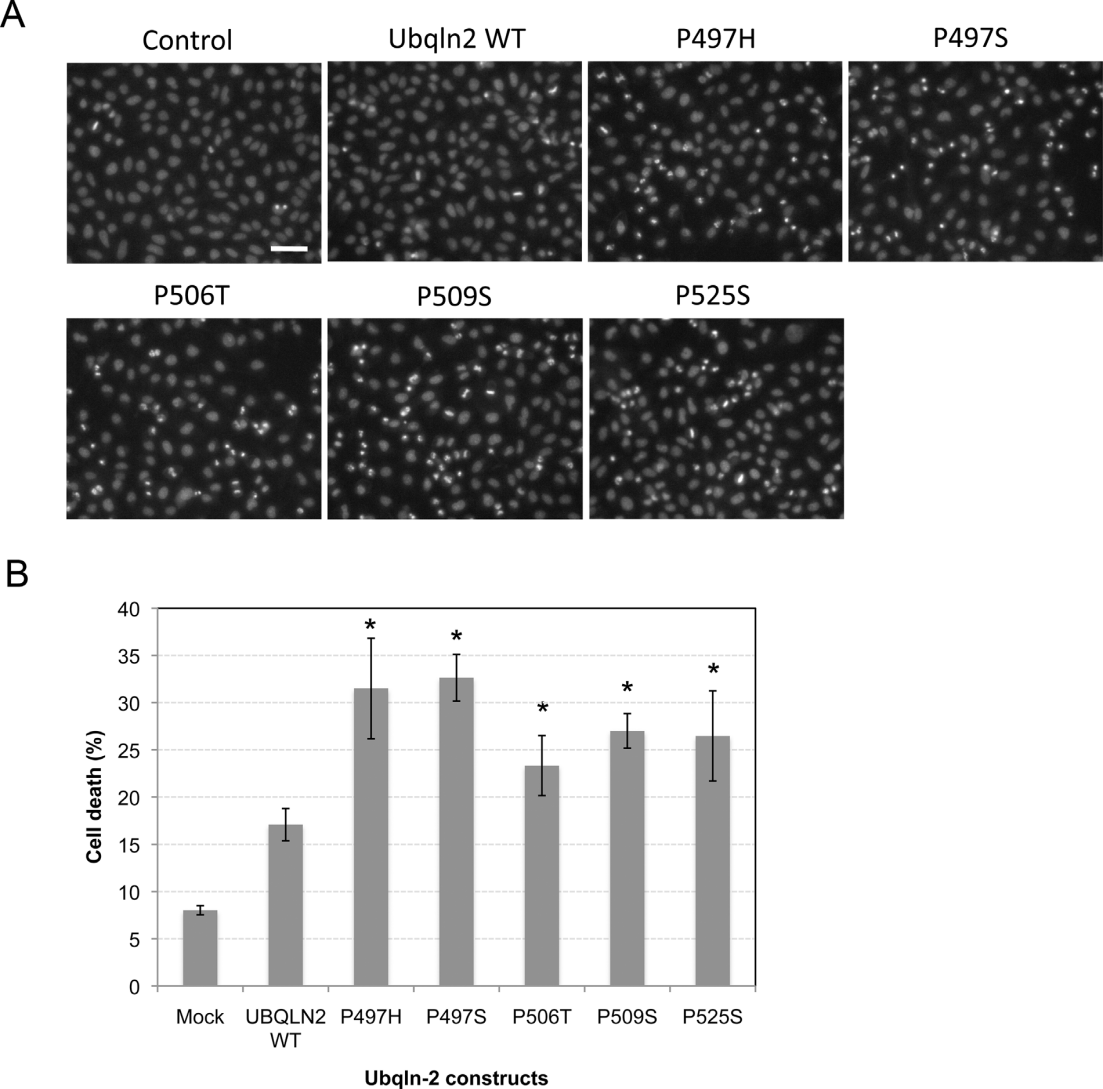
Overexpression of ubiquilin-2 proteins carrying ALS mutations increase cell death. HeLa cell cultures plated at similar density were either transfected with an empty vector (control), or with cDNAs encoding WT ubiquilin-2 or each of the five different ALS mutations. 24 after transfection the cells were stained with Hoechst and the expressed ubiquilin-2 proteins to identify dying cells and the transfection efficiency, respectively. The transfection efficiency in these experiments was >95% (not shown). Representative examples showing an increase in the proportion of cells that had bright abnormal nuclear morphology by Hoechst staining. Bar = 50 μm. (B) Graph represents the total number of cells that had abnormal bright nuclear morphology (excluding mitotic cells) as a percentage of the total number of cells. Errors bars show standard error of the mean. The * signifies a statistical increase in cell death compared with the mock transfected cells (p = <0.05).

A more plausible explanation for the detrimental effects of the mutants is that failure to deliver their captured ubiquitinated cargo would cause a buildup of ubiquitinated proteins in cells leading to dysregulation in protein degradation an increase in cell death. To examine this possibility we quantified cell death in HeLa cell cultures transfected with the different constructs (Fig 6). The quantification revealed that overexpression of all five mutants increased cell death significantly compared with the mock-transfected cells (Fig 6B, p<0.05). Overexpression of WT ubiquilin-2 also increased cell death, but the increase was smaller and was non-significant when compared to the mock-transfected cells.

## Discussion

Here we have presented evidence showing that ubiquilin-2 proteins carrying ALS-linked mutations slow degradation of Myc, a prototypic proteasome substrate. The retardation of protein degradation was found upon overexpression of all five ALS mutations we tested, but not upon overexpression of WT ubiquilin-2, demonstrating both the generality and specificity of the defect in only mutant ubiquilin-2 proteins. The interference in protein degradation is consistent with a similar defect reported previously for two of the mutants we studied [5, 10]. By examining differences in proteins that coimmunoprecipitated with WT and mutant ubiquilin-2 proteins we obtained insight into the likely mechanism by which the mutants impede protein degradation. Our results revealed that although ubiquilin-2 proteins containing the ALS mutations are competent in binding polyubiquitinated proteins they are less able to bind the proteasome compared with WT ubiquilin-2 protein. A likely explanation for the reduction in proteasome binding was our discovery that many of the mutant proteins bind weaker to the S5a subunit component of the19S cap of the proteasome compared with WT ubiquilin-2. The S5a subunit is a receptor to which the UBL domain of ubiquilin-2 normally docks [14]. Together these results suggest that the ALS mutations in ubiquilin-2 that we studied impede protein degradation by capturing, but failing to deliver the captured ubiquitinated cargo to the proteasome. The mechanism of inhibition is consistent with dominant interference of protein degradation by the mutants.

The reduction in binding of the ubiquilin-2 mutants to the proteasome that we uncovered through quantification of alpha 6 and S5a subunit binding was less obvious for some of the mutants. For example, the P497H mutant still displayed reasonable binding to the alpha 6 and S5a subunits in both our immunoprecipitation and GST-pulldown assays. Nevertheless, this mutant induced high accumulation of polyubiquitinated proteins in cells compared to the other mutants, clearly indicating it causes some defect in either inducing and/or clearing polyubiquitinated from cells. One possibility is that although the mutant can bind to both polyubiquitinated proteins and the proteasome, differences in its affinity in these binding could interfere with proteasome degradation. In this regard, it will be important to determine if the *UBQLN2* mutants have any alteration in their binding affinity with different ubiquitin chains and/or with other ubiquilin-2 receptors in the proteasome. A further possibility is that adventitious binding of the mutants with other proteins in the cell might also lead to a disruption in proteasome delivery. Investigation of these issues could be instructive.

The positions of the five ubiquilin-2 mutations we examined are all located in the central domain of the protein, close to, but not in the UBL domain itself. We suggest two possible mechanisms by which the mutations could reduce ubiquilin-2 interaction with the proteasome. First, either the amino acids mutated, or the sequence surrounding them, together with the UBL domain, contribute in proteasome binding. In this scenario loss of binding to the proteasome is directly related to an alteration in the binding sequence by the mutations in ubiquilin-2. A second possibility, and one that we favor, is that the mutations induce a structural change in the ubiquilin-2 polypeptide interfering with the ability of the UBL domain to bind the proteasome. Curiously all of the ALS mutations we studied are located closer to the UBA domain than the UBL domain, varying approximately 50 to 80 aa from the UBA domain and 390 to 420 aa from the UBL domain. Thus the mutations appear to have a more drastic effect on the more distal of its two functional domains. Obviously, we cannot rule out the possibility it affect both domains. We believe resolution into these and other possibilities will become evident once the structure of the WT and mutant ubiquilin-2 proteins are solved. This might require information when bound to their captured ubiquitinated cargo.

A prediction based on the reduced ability of the ubiquilin-2 mutants to bind the proteasome would mean that over time the captured ubiquitinated cargo bound to the mutants would accumulate in cells, potentially causing toxicity. Consistent with this idea, overexpression of the ubiquilin-2 mutants led to an overall increase in both cell death and accumulation of polyubiquitinated proteins in cells transfected with the majority of the mutants. A smaller increase was seen upon overexpression of the WT protein. A possible explanation for this increase is that excessive amounts of ubiquilin-2 in cells might lead to uncoordinated binding of the protein to ubiquitinated proteins and/or the proteasome, disrupting proteostasis. Another possibility is that overexpression could lead to unregulated binding of ubiquilin-2 to its other partners, such as erasin, UBXD8, hnRNPA1 or with other ubiquilin proteins, all of which have been shown to bind ubiquilin-2 [9, 18, 20]. Such unregulated binding could interfere with either one or both of these protein functions, leading to toxicity. Whether this toxicity is unique to overexpression of ubiquilin-2 or other ubiquilins remains to be established. It is interesting to note that we recently generated transgenic mice overexpressing WT ubiquilin-1 and found no detrimental effects from its overexpression [29].

Besides the defect in proteasomal degradation, a potentially compounding property of all the ALS mutations was their effect in altering the intrinsic stability of ubiquilin-2 protein itself. The turnover studies indicated that all the mutations examined, with the exception of the P497H mutation, significantly increased ubiquilin-2 protein stability. However, even the P497H mutation increased ubiquilin-2 protein to some degree. The implication of such a prolongation in their half-lives would mean that over time the mutant ubiquilin-2 proteins would increase in abundance in cells, thereby compounding toxicity.

The defect of the ubiquilin-2 mutants we studied here focused on its function in proteasome degradation. However, ubiquilin proteins have been implicated in several additional functions, including it more specialized role in ERAD as well as in autophagy [20, 30–32]. It will be important to examine if, and how, the mutations affect these or its other functions.

In summary our results have provided mechanistic insight into how certain ubiquilin-2 mutations interfere with protein degradation. The insight we have provided should be useful in devising therapeutic strategies to alleviate the toxicity.

## References

[1] Rowland LP, Shneider NA. Amyotrophic lateral sclerosis. N Engl J Med. 2001;344(22):1688–700. Epub 2001/06/02. 10.1056/NEJM200105313442207 . 11386269

[2] Ferraiuolo L, Kirby J, Grierson AJ, Sendtner M, Shaw PJ. Molecular pathways of motor neuron injury in amyotrophic lateral sclerosis. Nat Rev Neurol. 2011;7(11):616–30. Epub 2011/11/05. nrneurol.2011.152 [pii] 10.1038/nrneurol.2011.152 . 22051914

[3] Ling SC, Polymenidou M, Cleveland DW. Converging mechanisms in ALS and FTD: disrupted RNA and protein homeostasis. Neuron. 2013;79(3):416–38. Epub 2013/08/13. S0896-6273(13)00657-0 [pii] 10.1016/j.neuron.2013.07.033 . 23931993 PMC4411085

[4] Robberecht W, Philips T. The changing scene of amyotrophic lateral sclerosis. Nat Rev Neurosci. 2013;14(4):248–64. Epub 2013/03/07. nrn3430 [pii] 10.1038/nrn3430 . 23463272

[5] Deng HX, Chen W, Hong ST, Boycott KM, Gorrie GH, Siddique N, et al Mutations in UBQLN2 cause dominant X-linked juvenile and adult-onset ALS and ALS/dementia. Nature. 2011;477(7363):211–5. Epub 2011/08/23. nature10353 [pii] 10.1038/nature10353 21857683 PMC3169705

[6] Synofzik M, Maetzler W, Grehl T, Prudlo J, Vom Hagen JM, Haack T, et al Screening in ALS and FTD patients reveals 3 novel UBQLN2 mutations outside the PXX domain and a pure FTD phenotype. Neurobiol Aging. 2012;33(12):2949 e13–7. Epub 2012/08/16. S0197-4580(12)00377-6 [pii] 10.1016/j.neurobiolaging.2012.07.002 . 22892309

[7] Gellera C, Tiloca C, Del Bo R, Corrado L, Pensato V, Agostini J, et al Ubiquilin 2 mutations in Italian patients with amyotrophic lateral sclerosis and frontotemporal dementia. J Neurol Neurosurg Psychiatry. 2013;84(2):183–7. Epub 2012/11/10. jnnp-2012-303433 [pii] 10.1136/jnnp-2012-303433 . 23138764

[8] Fahed AC, McDonough B, Gouvion CM, Newell KL, Dure LS, Bebin M, et al UBQLN2 mutation causing heterogeneous X-linked dominant neurodegeneration. Ann Neurol. 2014;75(5):793–8. Epub 2014/04/29. 10.1002/ana.24164 . 24771548 PMC4106259

[9] Xia Y, Yan LH, Huang B, Liu M, Liu X, Huang C. Pathogenic mutation of UBQLN2 impairs its interaction with UBXD8 and disrupts endoplasmic reticulum-associated protein degradation. J Neurochem. 2013;129:99–106. Epub 2013/11/13. 10.1111/jnc.12606 . 24215460

[10] Gorrie GH, Fecto F, Radzicki D, Weiss C, Shi Y, Dong H, et al Dendritic spinopathy in transgenic mice expressing ALS/dementia-linked mutant UBQLN2. Proc Natl Acad Sci U S A. 2014;111(40):14524–9. Epub 2014/09/24. 1405741111 [pii] 10.1073/pnas.1405741111 25246588 PMC4209984

[11] Wu AL, Wang J, Zheleznyak A, Brown EJ. Ubiquitin-related proteins regulate interaction of vimentin intermediate filaments with the plasma membrane. Mol Cell. 1999;4(4):619–25. . 10549293 10.1016/s1097-2765(00)80212-9

[12] Mah AL, Perry G, Smith MA, Monteiro MJ. Identification of ubiquilin, a novel presenilin interactor that increases presenilin protein accumulation. J Cell Biol. 2000;151(4):847–62. . 11076969 10.1083/jcb.151.4.847 PMC2169435

[13] Kleijnen MF, Shih AH, Zhou P, Kumar S, Soccio RE, Kedersha NL, et al The hPLIC proteins may provide a link between the ubiquitination machinery and the proteasome. Mol Cell. 2000;6(2):409–19. . 10983987 10.1016/s1097-2765(00)00040-x

[14] Walters KJ, Kleijnen MF, Goh AM, Wagner G, Howley PM. Structural studies of the interaction between ubiquitin family proteins and proteasome subunit S5a. Biochemistry. 2002;41(6):1767–77. . 11827521 10.1021/bi011892y

[15] Ko HS, Uehara T, Tsuruma K, Nomura Y. Ubiquilin interacts with ubiquitylated proteins and proteasome through its ubiquitin-associated and ubiquitin-like domains. FEBS Lett. 2004;566(1–3):110–4. . 15147878 10.1016/j.febslet.2004.04.031

[16] Massey LK, Mah AL, Ford DL, Miller J, Liang J, Doong H, et al Overexpression of ubiquilin decreases ubiquitination and degradation of presenilin proteins. J Alzheimers Dis. 2004;6(1):79–92. . 15004330 10.3233/jad-2004-6109

[17] Ugolino J, Fang S, Kubisch C, Monteiro MJ. Mutant Atp13a2 proteins involved in parkinsonism are degraded by ER-associated degradation and sensitize cells to ER-stress induced cell death. Hum Mol Genet. 2011;20(18):3565–77. Epub 2011/06/15. ddr274 [pii] 10.1093/hmg/ddr274 21665991 PMC3159557

[18] Gilpin KM, Chang L, Monteiro MJ. ALS-linked mutations in ubiquilin-2 or hnRNPA1 reduce interaction between ubiquilin-2 and hnRNPA1. Hum Mol Genet. 2015;24:2565–77. Epub 2015/01/27. ddv020 [pii] 10.1093/hmg/ddv020 . 25616961

[19] Liang J, Yin C, Doong H, Fang S, Peterhoff C, Nixon RA, et al Characterization of erasin (UBXD2): a new ER protein that promotes ER-associated protein degradation. J Cell Sci. 2006;119(Pt 19):4011–24. Epub 2006/09/14. jcs.03163 [pii] 10.1242/jcs.03163 . 16968747

[20] Lim PJ, Danner R, Liang J, Doong H, Harman C, Srinivasan D, et al Ubiquilin and p97/VCP bind erasin, forming a complex involved in ERAD. J Cell Biol. 2009;187(2):201–17. Epub 2009/10/14. jcb.200903024 [pii] 10.1083/jcb.200903024 19822669 PMC2768832

[21] Monteiro MJ, Mical TI. Resolution of kinase activities during the HeLa cell cycle: identification of kinases with cyclic activities. Exp Cell Res. 1996;223(2):443–51. . 8601422 10.1006/excr.1996.0100

[22] Ford DL, Monteiro MJ. Studies of the role of ubiquitination in the interaction of ubiquilin with the loop and carboxyl terminal regions of presenilin-2. Biochemistry. 2007;46(30):8827–37. Epub 2007/07/07. 10.1021/bi700604q 17614368 PMC2547082

[23] Wu Q, Liu M, Huang C, Liu X, Huang B, Li N, et al Pathogenic Ubqln2 gains toxic properties to induce neuron death. Acta Neuropathol. 2014;129:417–28. Epub 2014/11/13. 10.1007/s00401-014-1367-y . 25388785 PMC4777328

[24] Rothenberg C, Monteiro MJ. Ubiquilin at a crossroads in protein degradation pathways. Autophagy. 2010;6(7):979–80. Epub 2010/08/24. 13118 [pii] 10.4161/auto.6.7.13118 . 20729634 PMC3359474

[25] Lee DY, Brown EJ. Ubiquilins in the crosstalk among proteolytic pathways. Biol Chem. 2012;393(6):441–7. Epub 2012/05/26. 10.1515/hsz-2012-0120/j/bchm.2012.393.issue-6/hsz-2012-0120/hsz-2012-0120.xml . 22628307

[26] Gregory MA, Hann SR. c-Myc proteolysis by the ubiquitin-proteasome pathway: stabilization of c-Myc in Burkitt's lymphoma cells. Mol Cell Biol. 2000;20(7):2423–35. Epub 2000/03/14. 10713166 10.1128/mcb.20.7.2423-2435.2000 PMC85426

[27] Voges D, Zwickl P, Baumeister W. The 26S proteasome: a molecular machine designed for controlled proteolysis. Annu Rev Biochem. 1999;68:1015–68. Epub 2000/06/29. 10.1146/annurev.biochem.68.1.1015 . 10872471

[28] Pickart CM, Cohen RE. Proteasomes and their kin: proteases in the machine age. Nat Rev Mol Cell Biol. 2004;5(3):177–87. Epub 2004/03/03. 10.1038/nrm1336nrm1336 . 14990998

[29] Safren N, El Ayadi A, Chang L, Terrillion CE, Gould TD, Boehning DF, et al Ubiquilin-1 overexpression increases the lifespan and delays accumulation of Huntingtin aggregates in the R6/2 mouse model of Huntington's disease. PLoS ONE. 2014;9(1):e87513 Epub 2014/01/30. doi: 10.1371/journal.pone.0087513 PONE-D-13-37940 24475300 10.1371/journal.pone.0087513 PMC3903676

[30] Kim TY, Kim E, Yoon SK, Yoon JB. Herp enhances ER-associated protein degradation by recruiting ubiquilins. Biochem Biophys Res Commun. 2008;369(2):741–6. Epub 2008/03/01. S0006-291X(08)00365-3 [pii] 10.1016/j.bbrc.2008.02.086 . 18307982

[31] N'Diaye EN, Kajihara KK, Hsieh I, Morisaki H, Debnath J, Brown EJ. PLIC proteins or ubiquilins regulate autophagy-dependent cell survival during nutrient starvation. EMBO Rep. 2009;10(2):173–9. Epub 2009/01/17. embor2008238 [pii] 10.1038/embor.2008.238 19148225 PMC2637314

[32] Rothenberg C, Srinivasan D, Mah L, Kaushik S, Peterhoff CM, Ugolino J, et al Ubiquilin functions in autophagy and is degraded by chaperone-mediated autophagy. Hum Mol Genet. 2010 Epub 2010/06/10. ddq231 [pii] 10.1093/hmg/ddq231 . 20529957 PMC2908472

